# Conditional Inactivation of *p53* in Mouse Ovarian Surface Epithelium Does Not Alter MIS Driven Smad2-Dominant Negative Epithelium-Lined Inclusion Cysts or Teratomas

**DOI:** 10.1371/journal.pone.0065067

**Published:** 2013-05-31

**Authors:** Suzanne M. Quartuccio, Daniel D. Lantvit, Maarten C. Bosland, Joanna E. Burdette

**Affiliations:** 1 Department of Medicinal Chemistry and Pharmacognosy, University of Illinois at Chicago, Chicago, Illinois, United States of America; 2 Department of Pathology, University of Illinois at Chicago, Chicago, Illinois, United States of America; Kinghorn Cancer Centre, Garvan Institute of Medical Research, Australia

## Abstract

Epithelial ovarian cancer is the most lethal gynecological malignancy among US women. The etiology of this disease, although poorly understood, may involve the ovarian surface epithelium or the epithelium of the fallopian tube fimbriae as the progenitor cell. Disruptions in the transforming growth factor beta (TGFβ) pathway and p53 are frequently found in chemotherapy-resistant serous ovarian tumors. Transgenic mice expressing a dominant negative form of Smad2 (Smad2DN), a downstream transcription factor of the TGFβ signaling pathway, targeted to tissues of the reproductive tract were created on a FVB background. These mice developed epithelium-lined inclusion cysts, a potential precursor lesion to ovarian cancer, which morphologically resembled oviductal epithelium but exhibited protein expression more closely resembling the ovarian surface epithelium. An additional genetic “hit” of *p53* deletion was predicted to result in ovarian tumors. Tissue specific deletion of *p53* in the ovaries and oviducts alone was attempted through intrabursal or intraoviductal injection of Cre-recombinase expressing adenovirus (AdCreGFP) into *p53* ^flox/flox^ mice. Ovarian bursal cysts were detected in some mice 6 months after intrabursal injection. No pathological abnormalities were detected in mice with intraoviductal injections, which may be related to decreased infectivity of the oviductal epithelium with adenovirus as compared to the ovarian surface epithelium. Bitransgenic mice, expressing both the Smad2DN transgene and *p53* ^flox/flox^, were then exposed to AdCreGFP in the bursa and oviductal lumen. These mice did not develop any additional phenotypes. Exposure to AdCreGFP is not an effective methodology for conditional deletion of floxed genes in oviductal epithelium and tissue specific promoters should be employed in future mouse models of the disease. In addition, a novel phenotype was observed in mice with high expression of the Smad2DN transgene as validated through qPCR analysis, characterized by teratoma-like lesions implicating Smad signaling in teratoma development.

## Introduction

Epithelial ovarian cancer is the most lethal cancer of the reproductive tract and fifth leading cause of cancer death among US women [1]. Most ovarian cancer patients are diagnosed only after the disease has metastasized into the peritoneal space because there are few early symptoms and no universally accepted precursor lesion. Subtle morphological changes in the ovary, including epithelium-lined inclusion cysts, may precede cancer formation [2] based on several pieces of evidence. First, serial sections of ovaries prophylactically removed from women with *BRCA* mutations, who are at high risk for developing ovarian cancer, often reveal metaplasia or hyperplasia in the cells lining these inclusion cysts [3]. Inclusion cysts are also commonly found in the ovary contralateral to a cancerous counterpart [4]. In addition, the cells lining inclusion cysts typically resemble Müllerian duct-derived epithelia [5], express CA-125 [6], [7], the serum biomarker for ovarian cancer, and increase in number as a woman ages.

The limited amount of “normal” and early stage ovarian tissue has impeded our understanding of the etiology of this disease including uncertainty in the cell type of origin, which may either be the ovarian surface epithelium (OSE) or epithelial cells of the fallopian tube fimbriae (TEC) [3], [8], [9]. Although the bulk of ovarian tumors are found within the ovary, high-grade serous ovarian cancers morphologically resemble the TEC. In addition, examination of prophylactic salpingo-oophorectomy samples from women with germline *BRCA* mutations revealed fallopian tube lesions but normal ovaries [10] suggesting a role for the fallopian tube in early ovarian cancer transformative events. Foci of strong immuno-staining for p53, known as the p53 signature, have been found in the otherwise benign, fimbriated end of the fallopian tube [8]. Many of these p53 signatures share identical *p53* mutations with high-grade serous ovarian tumors suggesting a common origin [11]. Regardless of origin, two recent meta-analyses of human microarray data both concluded that the pathways most frequently disrupted in platinum-based chemotherapy-resistant, high-grade serous ovarian cancer are p53 and transforming growth factor beta (TGFβ) [12], [13].

Although ovarian cancer is known to be a heterogeneous disease with various histological subtypes and molecular changes, the Cancer Genome Atlas Research Network reported that up to 96% of high-grade serous ovarian cancers, the most common and deadly histotype, contain mutations in *p53* [14]. The tumor suppressor p53 is implicated in many cellular processes including DNA repair, cell cycle arrest, and apoptosis, and it is the most frequently mutated gene in human cancers. Missense mutations in *p53* occur most often in the DNA binding domain. Mice with global deletion of *p53* spontaneously develop lymphomas and sarcomas at 6 months of age [15], before more slowly developing neoplasms like ovarian tumors can be formed or studied. Tissue specific loss-of-function *p53* mutations are generated by deletion of exons 2–10 in mice harboring the floxed gene. However, mice with this tissue specific deletion of *p53* alone in the ovarian surface epithelium have not been reported to develop serous ovarian cancers [16]–[18].

The TGFβ signaling pathway is involved in many cellular processes including proliferation, differentiation, and immune response. TGFβ acts as a tumor suppressor in normal and many early stage cancer cells. The transcription factors Smad2 and 3 are activated through phosphorylation by TGFβ type I receptor after ligand binding to the TGFβ type II receptor. These activated receptor-Smads (R-Smads) then oligomerize with the co-Smad, Smad4, and translocate to the nucleus where the complex binds DNA to influence gene transcription [19]. Mutations to TGFβ and its receptors are rare in ovarian cancer, but TGFβ pathway protein expression has been shown to be related to response to primary chemotherapy [13]. Disrupted TGFβ signaling was previously studied using a Smad2 dominant negative (Smad2DN) transgene driven by the Müllerian-inhibiting substance (MIS) promoter, targeting expression to cells of the reproductive tract in CD1 mice. In this model, the truncated Smad2 binds to the receptor but abrogates downstream signaling due to the lack of C-terminal serine residues that are normally phosphorylated and blocks wild-type Smad2 and 3 from binding to the receptor [20]. The CD1 Smad2DN mice developed endosalpingiosis, which has been implicated as a precursor for low grade serous ovarian cancer [21], and was characterized by epithelium-lined inclusion cysts that express cytokeratin 8 and morphologically resemble the oviduct (the murine equivalent to the fallopian tube) with secretory, ciliated, and pegylated cells which are seen in the normal oviduct [20]. This finding is of particular interest in light of the developing hypothesis of the TEC as a progenitor cell of some ovarian cancers. Since ovulation has been connected to ovarian cancer risk, a subsequent study superovulated the transgenic mice and found that although the number of inclusion cysts increased, they remained benign [22].

Development of an animal model of high-grade serous ovarian cancer is critical to understanding early transformative events, to identify precursor lesions and to elucidate the progenitor cell. In the present study the Smad2DN mice were analyzed to further characterize the cells lining the inclusion cysts. In addition, conditional inactivation of floxed *p53* genes in the OSE or TEC was then attempted through a direct comparison of viral exposure: the OSE is less differentiated than the TEC and exposure to viral components injected intrabursally is the only known method to target the OSE. Subsequently, the effects of *p53* gene inactivation combined with the Smad2DN transgene expression were investigated. Lastly, a novel teratoma-like tumor phenotype was reported in the Smad2DN mice with high transgene expression.

## Materials and Methods

### Ethics Statement

All animals were treated in accordance with the National Institutes of Health Guidelines for the Care and Use of Laboratory Animals and the established Institutional Animal Use and Care protocol at the University of Illinois at Chicago. The protocol was approved by the Animal Care Committee (protocol number: A08-250). Animals were housed in a temperature and light controlled environment (12 h light, 12 h dark) and were provided food and water *ad libitum*. All mice were euthanized by CO_2_ inhalation followed by cervical dislocation. Mice that developed tumors were euthanized when they had reached a loss-of-wellness endpoint due to tumor burden to minimize suffering.

### Regeneration of Smad2DN Transgenic Animals

The Smad2DN expression construct was a generous gift from Dr. Teresa Woodruff’s lab at Northwestern University and consists of a mouse minimal Müllerian-inhibiting substance (MIS) promoter (−180 bp), an epitope tag (Flag), a C-terminal truncation of the human Smad2 gene (dominant negative), and a human GH polyadenylation sequence. The Smad2DN plasmid was digested to liberate the transgene fragment, which was introduced into FVB fertilized oocytes through pronuclear microinjection by the University of Illinois at Chicago Transgenic Production Service. The Smad2DN transgene positive founder mouse was identified by Southern blot. All Smad2DN transgenic mice used in this study were derived from the same founder with the same number of transgene integrants. Tail samples were obtained from pups and genomic DNA was isolated by overnight digestion in a sodium chloride (NaCl)/EDTA/SDS/proteinase K solution at 55°C followed by salt precipitation and isopropanol/ethanol purification. PCR was performed using 32 cycles with an annealing temperature of 55°C. A 717 bp product was expected using the forward (5′ ACC ATG GAC TAC AAG GAC GAC 3′) and reverse (5′ ACT GAT ATA TCC AGG AGG TGG 3′) primers and Apex Taq polymerase (Genesee Scientific, San Diego, CA). Real time qPCR analysis of the transgene was performed using the forward (5′ ACC ATG GAC TAC AAG GAC GAC 3′) and reverse (5′ CTT TTC TTC CTG CCC ATT CTG 3′) primers. Equal concentrations of genomic DNA were added to FastStart Universal SYBR Green Master (ROX) (Roche, Indianapolis, IN) spiked with Apex Taq. The reaction was held at 95°C for ten minutes followed by 40 cycles of 95°C for 30 sec, 60°C for 45 sec, and 72°C for one minute. 18S was used as the reference gene and wild-type mice served as the negative control.

Normal mouse ovarian surface epithelium (MOSE), a kind gift from Dr. Barbara Vanderhyden, was isolated as previously described [23]. Cells were grown in media consisting of α-MEM (Mediatech Inc., Manassas, VA) supplemented with 10% v/v FBS (Gibco, Grand Island, NY), 2 mM L-glutamine (Gibco), 2 mg/ml EGF (Roche), 5 mg/ml insulin (Roche), 5 mg/ml transferrin (Roche), 5 ng/ml sodium selenite (Roche), and 1 mg/ml gentamycin (Mediatech) in 24 well plates at 50,000 cells per well until 70% confluent. The plasmid containing a Smad responsive element (SRE) fused to firefly luciferase reporter was a kind gift from Dr. Aris Moustakas [24] and was transfected into cells (50 ng/well) using TransIT LT1 transfection reagent (Mirus, Pittsburgh, PA) in Opti-MEM (Gibco) according to the manufacturer’s protocol. After transfection for 24 hours, cells were treated with TGFβ (10 ng/µl) (Sigma-Aldrich, St. Louis, MO) for 24 hours. The luciferase activity was measured as previously described [25].

### Floxed *p53* and Bitransgenic Animals

*p53* ^flox/flox^ [FVB;129-*Trp53* ^tm1Brn^] mice bearing LoxP in introns 2 and 10 of the *p53* gene were obtained from the Mouse Models of Human Cancers Consortium Mouse Repository (National Cancer Institute, Rockville, MD, USA). *p53* ^flox/flox^ mice were genotyped using the forward primer (5′ CAC AAA AAC AGG TTA AAC CAG 3′) and reverse primer (5′ AGC ACA TAG GAG GCA GAG AC 3′) to yield a 288 bp band for wild type or a 370 bp band for floxed sequences. The floxed *p53* mice were bred to homozygosity. These mice were then intercrossed through multiple generations to create a colony of bitransgenic mice (Smad2DN; *p53* ^flox/flox^).

### Adenovirus Administration

All attenuated adenovirus was purchased from Iowa Gene Vector Delivery Core (Iowa City, IA) at a concentration of 10^10^ plaque forming units (pfu)/ml. For surgical preparation, eight-week-old mice were anesthetized by intraperitoneal injection of 0.2 mL ketamine hydrochloride solution (Hospira, Inc., Lake Forest, IL) and xylazine hydrochloride, shaved and cleaned with betadine in a BSL2 lab. One midline dorsal incision was made followed by an incision into the peritoneal cavity above the fat pad of the right ovary. The ovary was externalized through the incision and intrabursal or intraoviductal injection was executed under microscopic magnification by inserting a 34-gauge beveled Hamilton (Reno, NV) needle through the ovarian fat pad into the ovarian bursa or into the luminal space of the oviduct to dispense a volume of 2 µL of adenovirus expressing Cre-recombinase and GFP (AdCreGFP). Only one ovary in each animal was injected, allowing the contralateral ovary to serve as the control. The tissue was then returned to the peritoneal cavity, inner incision sutured, and the outer incision was sealed with wound clips. For GFP and Cre localization, adenovirus was administered bilaterally and mice were euthanized 24 hours following injection. Confirmation of Cre-mediated *p53* recombination was determined through PCR amplification of DNA extracted from whole ovaries and oviducts intrabursally or intraoviductally injected with AdCreGFP 96 hours prior using primers previously described [17] to yield a 612 bp product.

Adenovirus infectivity was measured *in vitro* utilizing normal immortalized human ovarian surface epithelial cells (IOSE80, [26]), immortalized epithelial cells from the fallopian tube fimbriae of adult baboons (TEC40, [27]) and normal mouse oviductal epithelial cells isolated from CD1 mice (MTEC). The IOSE80 cells were maintained in 50% v/v Medium 199 (Gibco) and 50% v/v MCDB 105 (Sigma) with 15% v/v fetal bovine serum (FBS, Gibco), 2 mM L-glutamine (Gibco), 100 U/ml penicillin (Roche), 100 µg/ml streptomycin (Roche), and 5.5 µg/ml of EGF (Roche). The TEC40 cells were grown in MEM (Gibco) with 10% v/v FBS (Gibco), 2 mM L-glutamine (Gibco), 100 uM non-essential amino acids (Gibco), 1 mM sodium pyruvate (Gibco), 100 U/ml penicillin (Roche), and 100 ug/ml streptomycin (Roche). The MTEC cell line was prepared in our lab by physical dissociation of oviductal tissue from d16 CD1 mice. After 48–72 h the epithelial cells lining the oviduct migrated out of the tube and adhered to the tissue culture plastic. Spontaneous immortalization allowed for continuous passage in culture media consisting of α-MEM (Mediatech Inc., Manassas, VA) supplemented with 10% v/v FBS (Gibco), 2 mM L-glutamine (Gibco), 2 mg/ml EGF (Roche), 5 mg/ml insulin (Roche), 5 mg/ml transferrin (Roche), 5 ng/ml sodium selenite (Roche), 1 mg/ml gentamycin (Mediatech), and 18.2 ng/ml β-estradiol (Sigma-Aldrich). 50,000 cells of each cell line were plated into a chamber slide with 500 µL of the respective media. Cells were treated with 0.5 µl of AdCreGFP for 24 h, washed with PBS, DAPI stained, and coverslipped. Three random fields per well were counted to determine the percentage of cells infected.

### Preparation and Analysis of Tissues, Histology and Immunohistochemistry

The ovaries (with the ovarian fat pad and bursa intact) were removed along with the attached oviduct and a portion of the uterus, fixed in 4% paraformaldehyde (PFA) and paraffin-embedded. Five µm sections were cut for H&E staining, immunohistochemical (IHC), and immunofluorescence (IF) analysis as previously described [28]. In all cases, omission of the primary antibody was used as a negative control. The primary antibodies used in this study included 1∶100 rat monoclonal cytokeratin-8 TROMA-1 (Developmental Studies Hybridoma Bank, Iowa City, IA), 1∶1,000 mouse monoclonal acetylated tubulin (Sigma-Aldrich), 1∶500 rabbit polyclonal GFP (Cell Signaling Technology, Inc, Beverly, MA), 1∶50 rabbit polyclonal Pax8 (ProteinTech Group, Inc, Chicago, IL), 1∶250 rabbit polyclonal OVGP1 (Abcam, Cambridge, MA), 1∶50 rabbit monoclonal E-Cadherin (Cell Signaling Technology), 1∶200 rabbit polyclonal Ki67 (Abcam), 1∶25 goat polyclonal MIS (Santa Cruz Biotechnology, Inc, Santa Cruz, CA), 1∶500 rabbit polyclonal Cre (Abcam), and 1∶250 rabbit polyclonal phospho-Smad2/3 (Santa Cruz). All secondary antibodies were used at a concentration of 1∶200. For immunofluorescence (IF) goat, anti-rabbit Alexa Fluor 594 (Invitrogen, Eugene, OR) was used at a concentration of 1∶200. Stained sections were analyzed and classified by a laboratory animal pathologist (MCB).

### Data Analyses

All values are presented as the mean ± the standard error. ANOVA followed by Tukey’s multiple comparison tests were used to assess differences between experimental and control groups. P<0.05 was considered statistically significant.

## Results

### Mice with Low Smad2DN Transgene Expression Developed Epithelium-lined Inclusion Cysts, Which Followed a Staining Pattern Similar to Normal OSE

**Figure 1:**
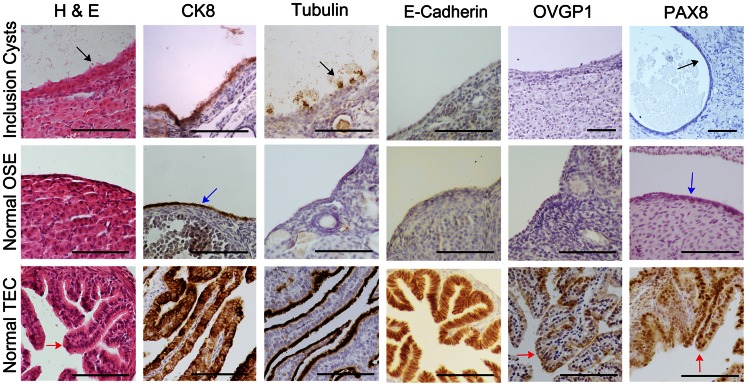
MIS-Smad2DN mice develop inclusion cysts with cells following a staining pattern similar to OSE. Ovaries removed from 3-month-old MIS driven Smad2DN transgenic mice were fixed, processed and analyzed. H&E stained tissue demonstrated that the cells lining the cysts morphologically resembled the normal TEC due to ciliation. Immunohistochemical analysis showed that cells lining the cysts expressed CK8 and acetylated tubulin but lacked expression of the normal oviductal markers E-Cadherin, OVGP1 and PAX8. Black arrows, blue arrows and red arrows indicate cells lining the inclusion cysts, OSE and TEC respectively. Scale bars represent 100 µm.

Previous work indicated that CD1 mice developed ovarian inclusion cysts lined with tubal/salpingeal epithelium (endosalpingiosis) when transgene expression for a dominant negative form of the TGFβ downstream transcription factor, Smad2, is driven by the Müllerian-inhibiting substance promoter (MIS) [20]. To further investigate these cysts with respect to the developing fallopian tube hypothesis as a potential source of ovarian cancer, the transgenic mice were created on the FVB background to allow for later crossbreeding with *p53* ^flox/flox^ mice while maintaining an inbred line. MIS expression was confirmed in the OSE and granulosa cells of secondary follicles (**Figure S1B**). The Smad2DN plasmid was verified to block Smad signaling when transfected into normal mouse ovarian surface epithelium in the presence of TGFβ (**Figure S1C**). Smad2DN mice, as determined through PCR amplification of the transgene (**Figure S1A**), displayed decreased phospho-Smad2/3 staining in the OSE (**Figure S1B**). Phospho-Smad2/3 staining was still observed in the granulosa cells of transgenic mice which agrees with previous reports that the transgene repression of Smad signaling was more evident in the OSE [20]. A similar inclusion cyst phenotype was observed on the FVB genetic background (**Figure 1**). The cells lining the cysts were epithelial, staining positive for cytokeratin 8 (CK8), and morphologically resembled the normal oviductal epithelium with ciliated cells (staining positive for acetylated tubulin). However, the cells lining the cysts lacked the expression of the epithelial cell adhesion molecule, E-Cadherin, oviductal epithelial cell marker, oviductal glycoprotein 1 (OVGP1), and the secretory cell marker, paired box 8 (PAX8). The staining pattern of the inclusion cysts was more similar to that of normal OSE than normal TEC.

### Injection of AdCreGFP into the Bursa of *p53* ^flox/flox^ to Target the OSE Resulted in Bursal Cysts and Degenerate Ovaries while no Change in Phenotype was Observed after Oviductal Injection

**Figure 2:**
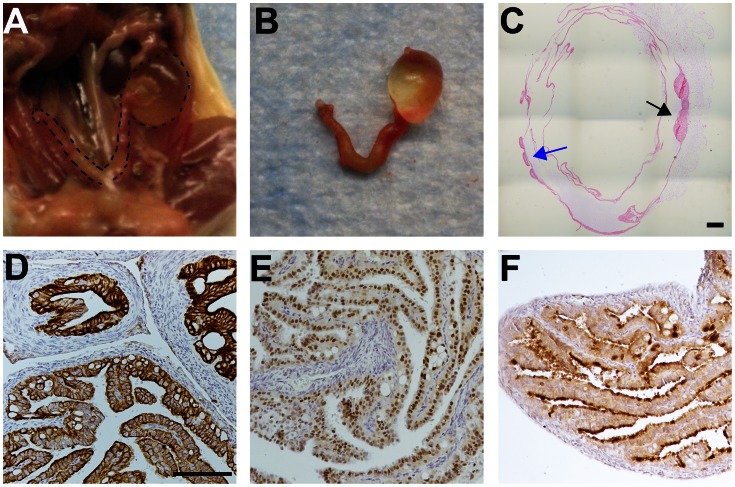
Intrabursal injection of AdCreGFP, but not intraoviductal injection, modifies ovarian phenotype. Fluid-filled bursal cysts and degenerate ovaries were observed in *p53* ^flox/flox^ mice 6 months after intrabursal injection of AdCreGFP (A-C). Black arrow indicates ovarian tissue and blue arrow indicates oviductal tissue. The TEC showed normal expression of CK8 (D), PAX8 (E), and OVGP1 (F) both three and six months after intraoviductal viral injection. Scale bar represents 100 µm.

An additional genetic “hit” might transform the inclusion cyst phenotype into cancer as other models of serous cancer have introduced multiple genetic alterations to generate tumors [29]. Deletion of *p53* was chosen because the tumor suppressor is mutated in approximately 96% of serous ovarian tumors [14]. Tissue specific deletion of *p53* alone was first characterized. To investigate both the ovarian surface and the oviductal epithelium as a potential site of origin, *p53* deletion via intrabursal or intraoviductal injection of AdCreGFP into *p53* ^flox/flox^ mice (**Figure S2A**) was attempted to target the OSE and TEC respectively. Mice subjected to intrabursal viral injections exhibited normal phenotypes 3 months after AdCreGFP injection (n = 13), while fluid-filled, bursal cysts and degenerated ovaries formed in two mice six months after injection (n = 7) (**Figure 2**). Intraoviductal injection of the AdCreGFP resulted in no change in phenotype after three months (n = 15). One *p53* ^flox/flox^ mouse subjected to intraoviductal AdCreGFP injection developed a fluid-filled, bursal cyst phenotype six months after infection (n = 5); however, viral leakage into the bursa was noted at the time of surgical injection and this phenotype closely mirrored that of intrabursal injections.

### Combining the Smad2DN Transgene Expression with AdCreGFP Injection into the Bursa and Oviductal Lumen of *p53* ^flox/flox^ mice did not Lead to an Additional Phenotype

Other animal models of ovarian cancer use multiple genetic manipulations. Bitransgenic animals were generated with both MIS-driven Smad2DN transgene and *p53* ^flox/flox^ expression that could be subjected to intrabursal or intraoviductal injection of AdCreGFP. Combining adenovirus infection of Cre-recombinase to delete *p53* with truncated TGFβ signaling due to MIS-Smad2DN expression did not lead to an altered ovarian or oviductal phenotype beyond Smad2DN expression or intrabursal injection of AdCreGFP alone (**Table 1**). Two Smad2DN; *p53* ^flox/flox^ mice developed a novel teratoma-like phenotype three months following intrabursal injection of AdCreGFP (n = 6), which was attributed to high Smad2DN transgene expression and was validated through real time qPCR. One Smad2DN; *p53* ^flox/flox^ developed a fluid-filled, bursal cyst six months after intrabursal injection of AdCreGFP (n = 7). The Smad2DN; *p53* ^flox/flox^ mice with intraoviductal injection of AdCreGFP exhibited no additional ovarian or oviductal phenotypes (n = 11 at 3 months; n = 6 at 6 months).

**Table 1:** Summary of Tumor Incidence.

Genotype	Injection Site	Latency Period	Mice with Phenotype (%)
*p53* ^flox/flox^	Bursa	3 months	0/13
	Bursa	6 months	2/7 (29) Bursal Cyst
	Oviduct	3 months	0/15
	Oviduct	6 months	1/5*
Smad2DN; *p53* ^flox/flox^	Bursa	3 months	2/6 (33) Teratoma
	Bursa	6 months	1/7 (14) Bursal Cyst
	Oviduct	3 months	0/11
	Oviduct	6 months	0/6
Smad2DN^+/+^	N/A	2–6 months	5/18 (28) Teratoma

* Intraoviductal injection with leakage into the bursa.

### TEC was Less Susceptible to Adenoviral Infection Compared to OSE *in vivo*

**Figure 3:**
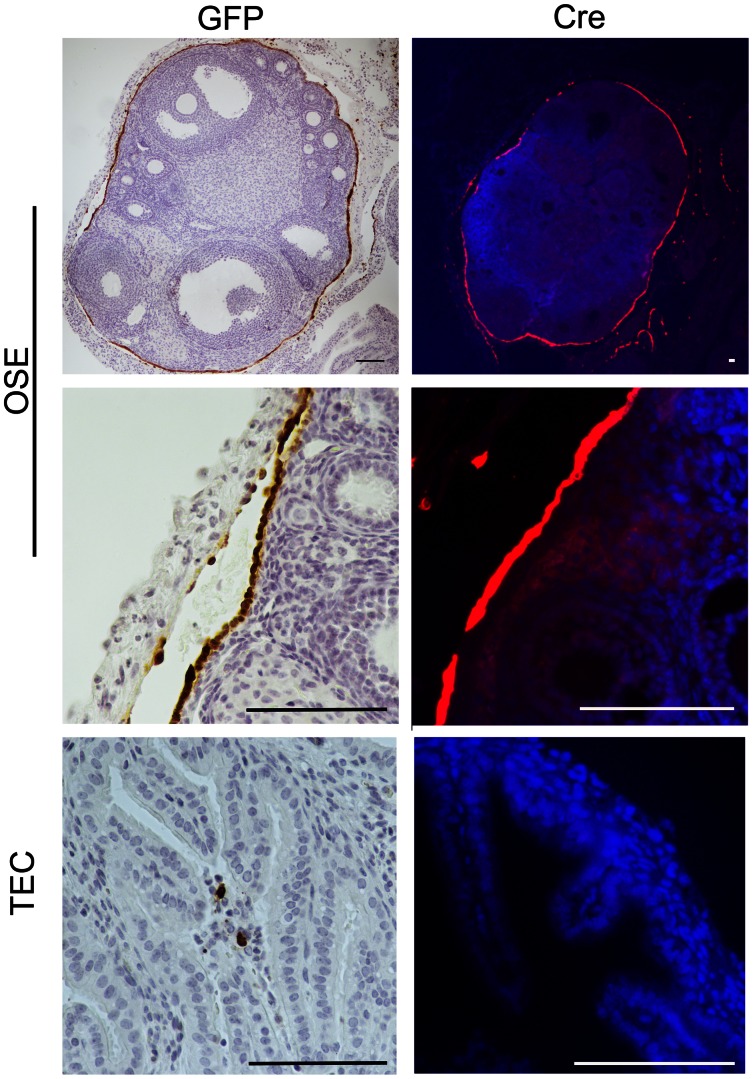
Intrabursal or intraoviductal injection of adenovirus results in OSE or TEC specific infection. Immunohistochemical and immunofluorescent detection of GFP and Cre-recombinase indicates tissue specific infection. Intrabursal injection shows staining of the OSE with limited leakage to adjacent tissue including the bursa. Intraoviductal injection results in limited infection of the TEC. Scale bars represent 100 µm.

Due to the lack of tissue specific promoters, adenovirus infection through intrabursal administration has previously been used as a method for effective infection of the OSE [17], [30], [31], but it was unknown if oviductal injections of adenovirus could similarly infect the TEC. To investigate the adenoviral infection of the OSE and TEC, animals were subjected to intrabursal or intraoviductal injection of AdCreGFP and the reproductive tract was removed 24 hours following surgical injection to analyze virus localization. IHC analysis for GFP and IF analysis for Cre expression in fixed tissue demonstrated that intrabursal administration infects the OSE without penetrating into the ovarian stroma. It should be noted that some bursal cells were also infected by this method and stained positive for GFP and Cre **(** **Figure 3** **)**. However, intraoviductal injection resulted in the infection of only a few TEC. Recombination of the loxP site was confirmed in genomic DNA from uterine cells infected *ex vivo* (positive control) and injected ovaries, however, injected oviducts and non-injected negative controls showed no recombination 96 hours following AdCreGFP introduction (**Figure S2B**). In contrast, chamber slide immunocytochemistry of GFP from viral infection in cell culture resulted in a similar percentage of oviductal epithelial cells infected with adenovirus as compared to ovarian surface epithelial cells *in vitro* (**Figure S3**).

### Mice with High Smad2DN Transgene Expression Developed a Novel Teratoma-like Phenotype

**Figure 4:**
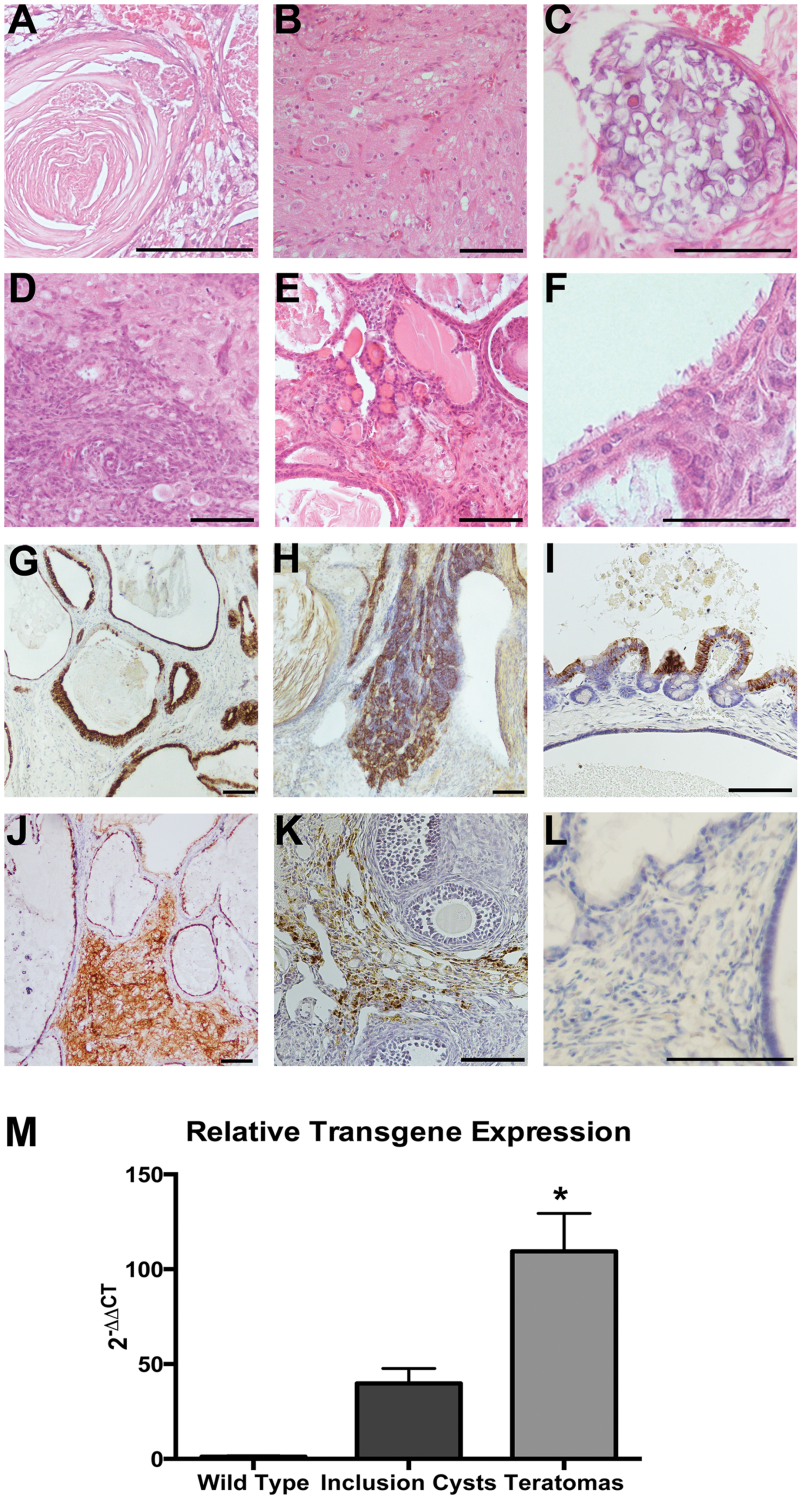
High Smad2DN transgene expression on a FVB background results in teratoma formation. The large teratomas contained many diverse structures including squamous cysts filled with layers of keratin (A), nervous tissue (B), bone formation (C), undifferentiated cells invading into an area of nervous tissue-like cells (D), thyroid follicle-like glands (E), and ciliated cells lining fluid-filled cysts (F). Immunohistochemical staining for CK8 was strongly positive in epithelial cells lining inclusion cysts (G) and infiltrating tumor cells (H). The OSE had an altered, papillary morphology and stained positive for epithelial marker CK8 (I). Acetylated tubulin marked the ciliated cells of the inclusion cysts as well as ovarian stroma (J). Abnormal OVGP1 expression was noted in the ovarian cortex obtained from an eight-week-old mouse (K). The teratomas lacked staining for phopsho-Smad2/3 (L). Scale bar represent 100 µm**.** Amplification of the Smad2DN by real time qPCR revealed significantly higher transgene expression in mice with the teratoma phenotype as compared to the mice with only the ovarian inclusion cysts phenotype (M).

An unexpected ovarian phenotype classified as mature teratomas, some of which appeared morphologically malignant, developed in five out of 15 (33%) mice with high transgene expression (as determined through real time qPCR). The teratomas were found in mice as early as eleven weeks of age and were characterized by areas of invasive, undifferentiated cells and inclusion cysts lined with ciliated cells reminiscent of oviductal or respiratory epithelium (**Figure 4A**). Three of the tumors also contained squamous cell-lined cysts filled with keratin in addition to well-to-moderately differentiated invasive squamous cells and nervous tissue-like areas. Fat, blood-filled spaces, calcified bone and cartilage formation, and thyroid gland-like structures were noted in some of these teratomas. By six months of age, the teratomas were roughly ten times the size of a normal ovary, did not display normal follicular or ovarian structures, and contained many epithelium-lined inclusion cysts that were highly proliferative as evidenced by positive Ki67 staining (data not shown). The CK8 staining of the OSE and cells lining the cysts was sporadic possibly indicating that the keratinization process of these cells was not complete. Ciliated cells, indicated by positive acetylated tubulin staining, were abundant in cells lining the inclusion cysts and in the ovarian cortex whereas secretory cells, indicated by positive PAX8 staining, were less common (data not shown).

Three additional mice with high transgene expression exhibited abnormal tumor-like ovaries at eight weeks of age. These tumors exhibited some, but not all of the characteristics of the mature teratomas observed at 6 months and could represent precursor lesions. Large nucleated cells were noted that resembled degenerative oocytes, although regions of normal ovarian structure including developing follicles and corpus lutea were also observed. Abnormal OVGP1 staining in the ovarian cortex was noted in at least one tumor from an eight-week old mouse. The teratomas showed a lack of phopho-Smad2/3 staining and real time qPCR revealed a significant almost three-fold higher transgene expression in mice with the ovarian teratomas than in mice with only inclusion cysts (**Figure 4B** **).** None of the wild-type or mice with low transgene expression developed these tumors.

## Discussion

Mice with MIS promoter-driven expression of a Smad2DN transgene developed epithelium-lined inclusion cysts that morphologically resembled the oviduct with respect to ciliation and acetylated tubulin expression but lacked E-Cadherin, OVGP1, and PAX8 expression, which are all absent from normal OSE. Intrabursal injection of some mice with adenovirus expressing Cre-recombinase to delete floxed *p53* in the OSE resulted in degenerated ovaries with large fluid-filled, bursal cysts. In contrast, intraoviductal injection did not result in any discernible phenotype, which could be attributed to poor infection of oviductal epithelium. The attempt to combine Smad2DN expression with tissue specific *p53* deletion did not generate any additional phenotype. High levels of Smad2DN transgene expression on the FVB background led to the development of mixed cell tumors with morphological features consistent with teratoma, which was previously not observed in transgenic mice on the CD1 background [20].

FVB mice expressing a MIS-driven Smad2DN transgene developed an inclusion cyst phenotype, as was previously reported in CD1 mice [20]. The cells lining the cyst were epithelial, staining positive for cytokeratin 8. Cell morphology and expression of acetylated tubulin were similar to normal TEC but the negative E-Cadherin, OVGP1, and PAX8 staining was similar to that of normal OSE. It has been proposed that the uncommitted OSE undergo Müllerian metaplasia before becoming carcinoma in a stepwise manner [32]. Since the cells lining the cysts examined here display both normal OSE and TEC markers it is possible that they were derived from the OSE and the Müllerian differentiation process is not complete. Alternatively, the cysts may be of oviductal origin [21] and have lost expression of TEC markers due to exposure to hormones and growth factors in the ovarian stroma microenvironment. These results are in agreement with human ovarian tumor samples, which also show markers for both ovarian surface and oviductal epithelium [33].

Intrabursal injection of AdCreGFP resulted in the development of fluid filled bursal cysts and degenerate ovaries in some mice six months following injection. GFP and Cre staining were used as surrogate markers for localization of virally infected cells. Conditional deletion of floxed genes in the OSE via intrabursal injection of AdCre is a commonly used method in the field since the OSE are less differentiated than other cells derived from the coelomic epithelium and lack tissue specific promoters [34]. Previous reports of *p53* inactivation through intrabursal injection of AdCre into floxed animals showed successful excision of LoxP flanked sequences and reported leiomyosarcomas formation [16], [17] or no ovarian phenotype at all [18]. These discrepancies can be attributed to the amount of adenovirus injected, whether the injections were unilateral or bilateral, latency period or slight variations in injection strategy [35]. Intrabursal injections can infect the cells of the bursal sac surrounding the mouse ovary and were the suggested source of the leiomyosarcomas observed. No study has reported the formation of epithelial ovarian cancer from conditional *p53* deletion alone. These data suggest that *p53* inactivation is not the initiating event in ovarian cancer, however it may contribute to disease promotion and progression.

This study is the first to report on intraoviductal injections of AdCreGFP as a means to conditionally inactivate floxed genes specifically in the TEC and identified no oviductal phenotype. The lack of a phenotype from *p53* deletion could be due to poor adenoviral infection into oviductal cells. Large volume injections into the bursa are known to leak to adjacent tissue including the oviduct and even the uterus, but no [17], [36] or very few (11/537) oviductal transformed phenotypes [18] have been reported. This observation supports the hypothesis that the TEC are less sensitized to adenovirus infection [35]. Our lab previously reported that the TEC has a lower proliferation rate as compared to the OSE in response to ovulation [27], which could limit transcription of Cre-recombinase needed for *p53* inactivation and viral infection. While the *in vitro* data presented here suggests that the TEC can be infected with adenovirus as well as OSE, there is *in vivo* evidence that TEC may be poised to respond to viral infection through toll-like receptors mediating an inflammatory response to inhibit viral expression [37]. Also, the level of the viral receptors (CAR) and its co-regulators (αvβ3 or αvβ5 integrins) determine the cell infection level and should not be assumed to be equal in OSE and TEC [35]. An oviductal phenotype was reported when the ALK5 receptor was conditionally deleted from mice with MIS receptor II (MISRII) promoter driven expression of Cre-recombinase, which resulted in oviductal diverticula but no ovarian phenotype [38]. The tissue specific expression of MIS and MISRII are not identical and the total deletion of the ALK5 receptor instead of the introduction of a dominant negative Smad protein likely account for the different phenotypes observed. The use of tissue specific promoters to drive Cre expression would overcome the shortcomings of poor viral infectivity of the TEC.

The combined expression of Smad2DN transgene and *p53* inactivation did not lead to an additional phenotype beyond Smad2DN expression alone. It is probable that the two genetic hits did not occur in the same cell at the same time [29], as the MIS-driven Smad2DN inclusion cysts develop spontaneously between three and six months of age and the adenovirus expression is limited to about 14–21 days following injection [39] and tumor formation does not occur unless immortalization and unchecked proliferation are achieved. Simultaneous expression could be achieved by using the MIS promoter to drive Cre-recombinase expression in the OSE, but expression of Cre would also occur in non-OSE cells such as granulosa cells precluding definitive characterization of the source of any tumors formed.

Mature benign cystic teratomas, or dermoid cysts, are tumors derived from all three germ-layers and comprise 20–35% of all human ovarian neoplasms [40]. Malignant conversion of teratomas is rare in humans (2% of all teratomas) and most commonly results in squamous cell carcinoma [41]. Some of the teratomas observed in this study had morphologic features indicative of malignancy, such as locally invasive, undifferentiated squamous cells, but invasion into tissues beyond the ovary was not observed. The teratomas most likely developed from loss of TGFβ signaling through Smad2DN transgene expression in follicular cells. It is well established that TGFβ is needed to maintain pluripotency in human embryonic stem cell populations [42]. Alterations in TGFβ signaling has been reported in teratocarcinoma development *in vitro* and *in vivo* [43] and the mouse teratoma-derived cell line showed increased tumorigenic properties with decreased TGFβ response [44]. While further studies are needed to elucidate the mechanism of teratoma formation, attenuated TGFβ signaling in the granulosa cells or developing follicles could alter the microenvironment of oocytes through paracrine signaling leading to teratoma formation, as seen in a recent publication involving Hedgehog signaling [45].

A mouse model of ovarian cancer would yield better understanding of early transformative events, help identify precursor lesions, and allow for testing of therapeutics and chemopreventative agents *in vivo.* The model presented here demonstrates that high expression of Smad2DN can induce teratomas, while low expression is associated with the formation of ovarian inclusion cysts that have morphological features of both the OSE and TEC. This study also investigated conditional *p53* deletion via intraoviductal and intrabursal injection of AdCreGFP and identified that tubal epithelial cells are less likely to be infected with adenovirus *in vivo* as compared to the ovarian surface epithelium. The attempt to combine expression of Smad2DN and conditional *p53* deletion did not lead to additional phenotypes. Thus, the development of an oviductal epithelium specific Cre-recombinase model is needed to test the importance of these cells as the source of serous ovarian cancer.

## Supporting Information

Figure S1 Genotyping analysis of DNA isolated from Smad2DN (+) and WT (−) mice (A). MIS is expressed in normal OSE and granulosa cells of secondary follicles. Smad2DN mice exhibit less phospho-Smad2/3 expression in the OSE compared to normal littermates (B). Transfection of the Smad2DN plasmid into MOSE cells reduces responsiveness to TGFβ (C).
(TIF)

Figure S2 **Genotyping analysis of DNA isolated from mice with wild type** ***p53*** **(WT), floxed** ***p53*** **(Flox) or both (Het).** Only mice with homozygous expression of the floxed allele were used in this study (A). A 612 bp fragment was produced using primers previously described [17] to confirm recombination of the loxP site and presence of *p53* ^Δ2–10^ in injected ovarian tissue while a lack of recombination is evident in injected oviductal tissue. DNA extracted from primary uterine cells obtained from *p53* ^flox/flox^ animals infected with AdCreGFP *ex vivo* served as the positive control while DNA from tissue not injected served as the negative control (B).
(TIF)

Figure S3 **Adenoviral infection of normal OSE and TEC cell lines.** Mouse CD1 (MTEC) and baboon (TEC40) oviductal epithelial cell lines infect as well as human ovarian surface epithelial cells (IOSE80) with adenovirus *in vitro*.
(TIF)
