# Supplementary figures and images for: Conditional Inactivation of p53 in Mouse Ovarian Surface Epithelium Does Not Alter MIS Driven Smad2-Dominant Negative Epithelium-Lined Inclusion Cysts or Teratomas
Source: PLoS One. 2013 May 31;8(5):e65067. doi: 10.1371/journal.pone.0065067 (PMC3669126; doi:10.1371/journal.pone.0065067)

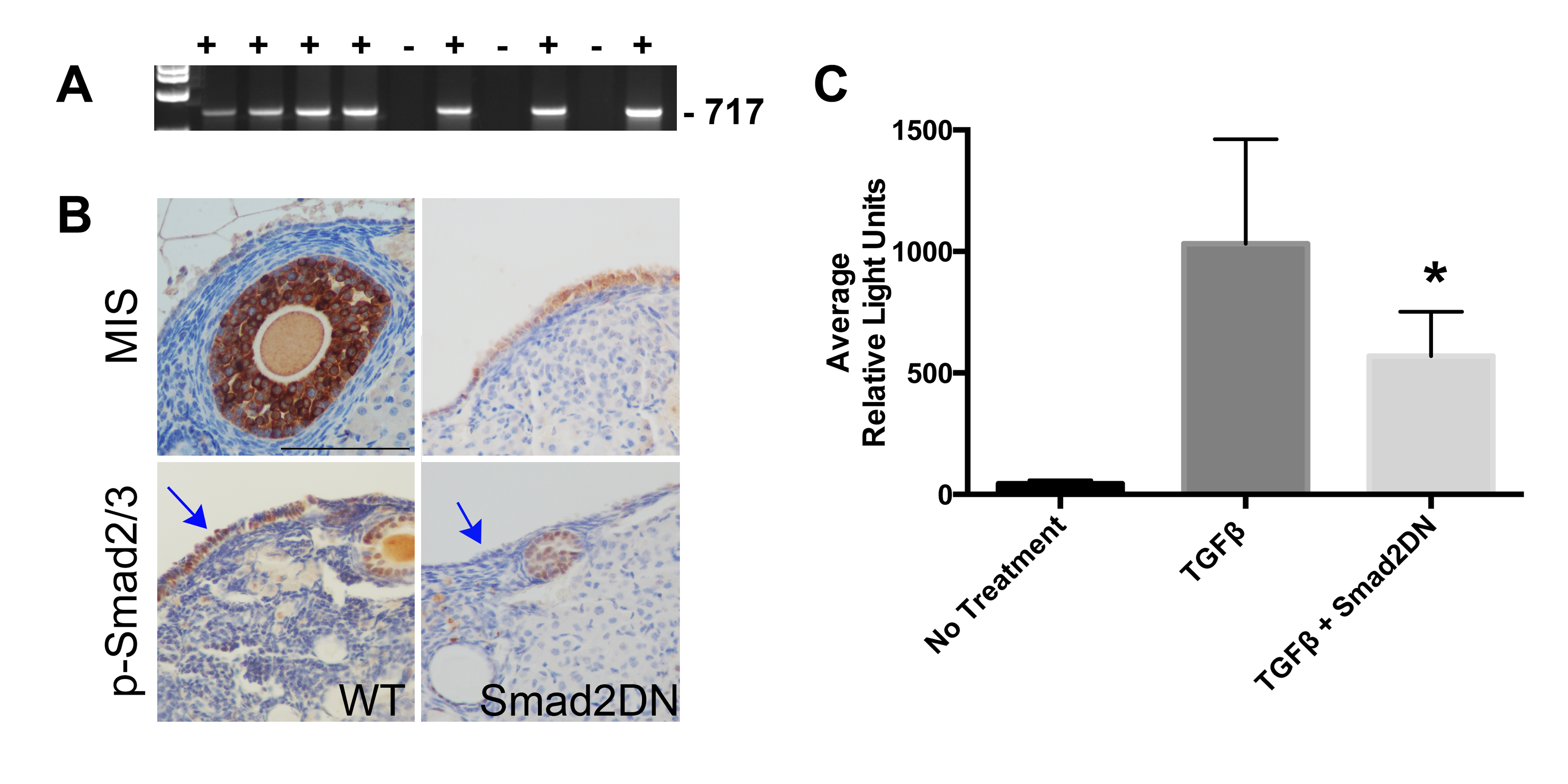

Supplement: Figure S1 — Genotyping analysis of DNA isolated from Smad2DN (+) and WT (−) mice (A). MIS is expressed in normal OSE and granulosa cells of secondary follicles. Smad2DN mice exhibit less phospho-Smad2/3 expression in the OSE compared to normal littermates (B). Transfection of the Smad2DN plasmid into MOSE cells reduces responsiveness to TGFβ (C). (TIF) [file pone.0065067.s001.tif]

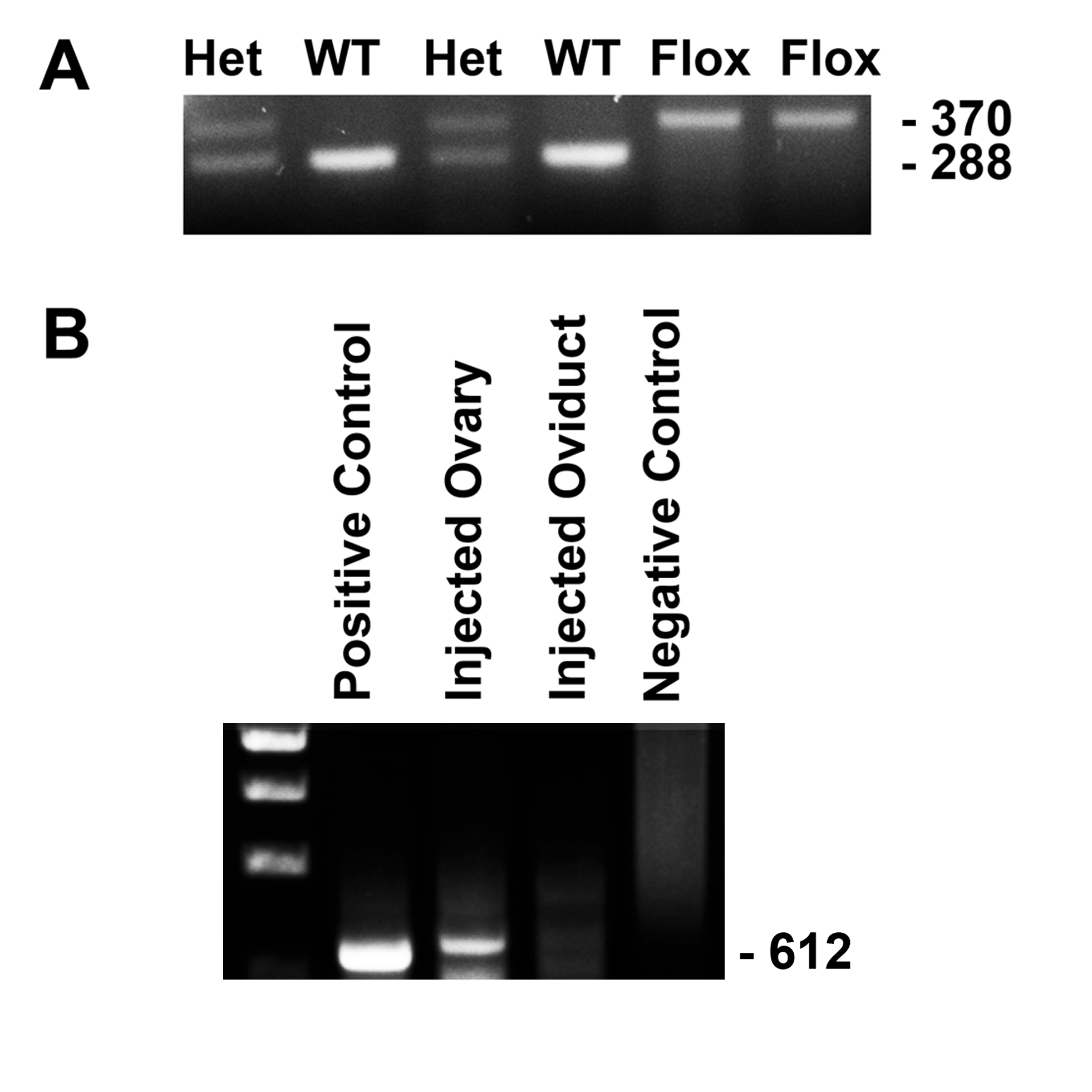

Supplement: Figure S2 — Genotyping analysis of DNA isolated from mice with wild type p53 (WT), floxed p53 (Flox) or both (Het). Only mice with homozygous expression of the floxed allele were used in this study (A). A 612 bp fragment was produced using primers previously described [17] to confirm recombination of the loxP site and presence of p53 Δ2–10 in injected ovarian tissue while a lack of recombination is evident in injected oviductal tissue. DNA extracted from primary uterine cells obtained from p53 flox/flox animals infected with AdCreGFP ex vivo served as the positive control while DNA from tissue not injected served as the negative control (B). (TIF) [file pone.0065067.s002.tif]

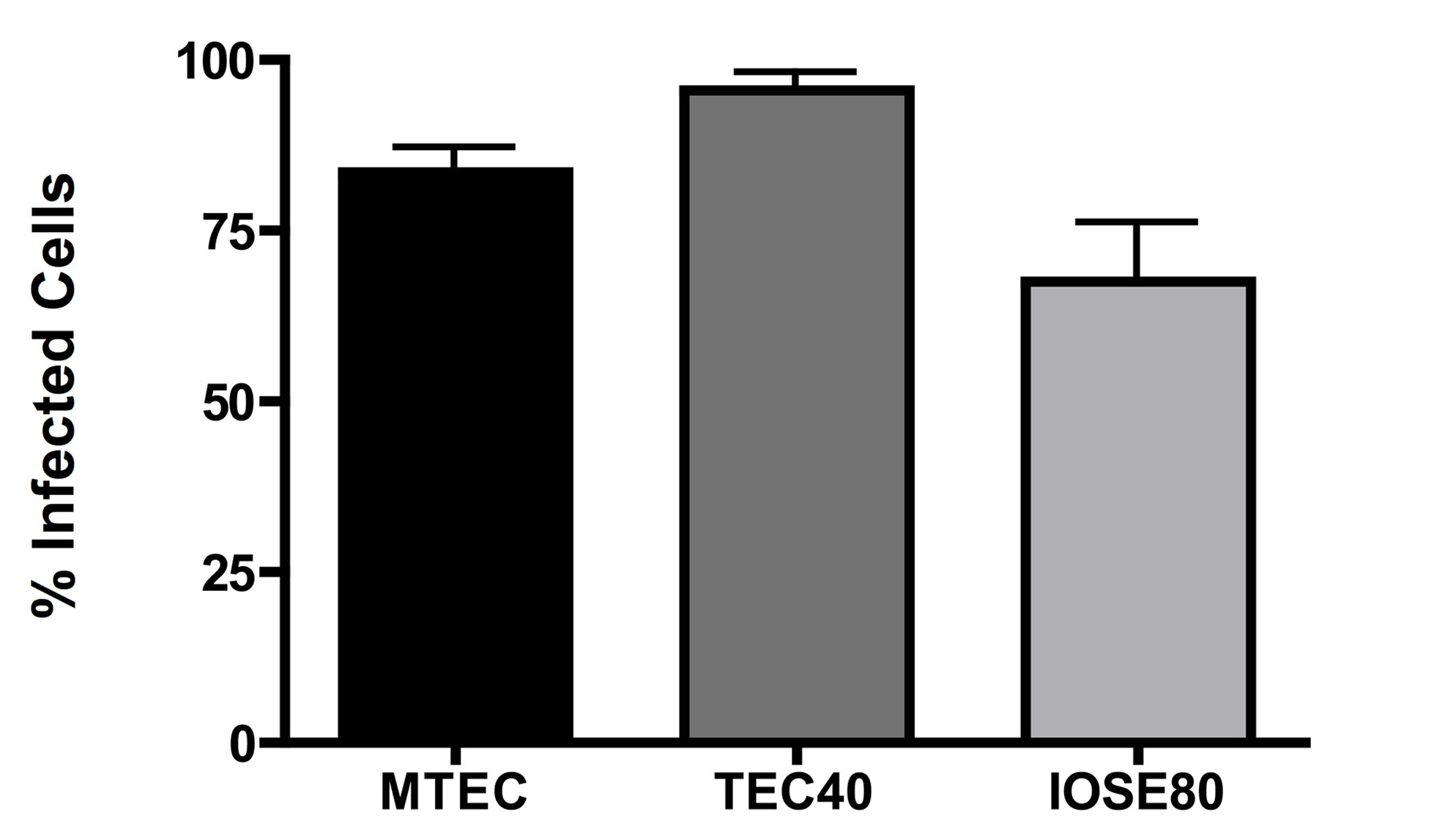

Supplement: Figure S3 — Adenoviral infection of normal OSE and TEC cell lines. Mouse CD1 (MTEC) and baboon (TEC40) oviductal epithelial cell lines infect as well as human ovarian surface epithelial cells (IOSE80) with adenovirus in vitro. (TIF) [file pone.0065067.s003.tif]
